# Pembrolizumab as a promising intervention for advanced penile cancer

**DOI:** 10.1590/S1677-5538.IBJU.2022.99.17

**Published:** 2022-04-10

**Authors:** Daniela Vinueza-Obando, Philippe E. Spiess, Herney Andrés García-Perdomo

**Affiliations:** 1 School of Medicine Universidad del Valle Cali Colombia UROGIV Research Group. School of Medicine. Universidad del Valle. Cali, Colombia;; 2 Department of Genito-Urinary Oncology H. Lee Moffitt Cancer Center Research Institute Tampa FL USA Department of Genito-Urinary Oncology, H. Lee Moffitt Cancer Center and Research Institute, Tampa, FL, USA;; 3 Department of Urology and Oncology University of South Florida Tampa FL USA Department of Urology and Oncology, University of South Florida, Tampa, FL, USA;; 4 Department of Surgery School of Medicine Universidad del Valle Cali Colombia Division of Urology/Urooncology. Department of Surgery, School of Medicine. Universidad del Valle. Cali, Colombia

## COMMENT

Penile cancer (PeCa) as a rare neoplasm has an incidence of 0.1 to 0.9 per 100,000 men in Europe and the USA. Some factors related to this epidemiologic difference include HPV infection status, smoking history, poor hygiene, and lack of infant circumcision. Most patients show an initial period of local growth, followed by regional node compromise and, finally, distant spread. Unfortunately, patients who show at advanced stages have a grim prognosis. Studies have shown one-third of patients who have regional recurrences are alive at five years, and none with distant metastases live longer than two years ( 1 , 2 ).

Standard treatments used in penile cancer patients with recurrence and metastatic disease include schemes with paclitaxel, ifosfamide, and cisplatin (TIP). Disappointingly, the efficacy of these agents has been recently contested ( 3 ) and overall survival rates do not exceed twelve months ( 2 ). Since its approval in 2014 ( 4 ) and its further indication as salvage therapy in certain penile SCC ( 5 ), pembrolizumab has been considered as a relevant therapeutic option.

Considering that there are no clinical trials to guide systemic therapy recommendations, we aimed to discuss the effectiveness and safety of pembrolizumab in patients with locally advanced or metastatic penile SCC.

When searching the vast literature through most databases, we found scarce information regarding this topic. Only two studies accomplished this criteria: Hahn et al. ( 6 ) and Chahoud et al. ( 7 ).

Regarding the general characteristics of people requiring immunotherapy, we might highlight that they are usually older patients with advanced stage penile cancer. Patients commonly show mass sensation, non-healing penile lesions, bloody discharge, and inguinal lymphadenopathies. Furthermore, they have T2-3 disease, N0-3, recurrent or even metastatic, squamous cell carcinoma (SCC) with a moderate to poor differentiation.

Consequently, patients undergo a multimodal therapy. A partial or radical penectomy, and bilateral and pelvic lymph node dissection are their initial and stepped surgical approach. Consolidation surgery may comprise a wide hemipelvectomy resection with acetabular reconstruction. Among patients, commonly used chemotherapeutic schemas included cisplatin/gemcitabine/ifosfamide and paclitaxel/ifosfamide/cisplatin, and they also use radiation therapy.

Although, patients may share interesting features regarding the biomarker expression, these are heterogeneous. PD-L1 expression and tumor mutational burden (TMB) are present in almost all patients. Moreover, tumor proportion score (TPS) is around 10%, and there is a combined positive score (CPS) of 1, 80 and 130. Furthermore, microsatellite instability (MSI) might be stable or high, and the tumor-infiltrating score (TIL) score may be consistent with few and moderate lymphocytic infiltration. Finally, there might be between three and 14 mutations per mega-base; however, there is no report of mismatch repair deficiency. There are other molecular alterations found using Foundation One that might be essential for future analysis ( Supplementary Table-1 ).

**Supplementary Table 1:** Molecular disturbances.

PTCH1 S1203fs*52 (VAF 19.2%)
EP300 N419fs*12 (VAF 20.3%)
FAT1 S1669* (VAF 33.1%)
HSD3B1 G171R (VAF 1.2%)
MLL2 L4921fs*74 (VAF 21.9%)
MLL2 P2354fs*30 (VAF 22.9%)
QKI K134fs*14 (VAF 24.4%)
MYD88 L265P (VAF 1.5%)
NFE2L2 W24R (VAF 36.4%)
SMARCA4 M1233I (VAF 8.9%)
TERT promoter 146C>T (VAF 18.7%)
TP53 R280G (VAF 18.3%)

Specifically, for pembrolizumab usage, we found that patients receive between two and nine cycles with a total time on treatment of 1.7 to 8.1 months ( 6 ). Authors also reported adverse effects such as Grade 2 hypothyroidism, maculopapular rash, and anorexia ( 6 ), and hypothyroidism ( 7 ).

Pembrolizumab use must follow the RECIST 1.1 criteria to evaluate the outcomes. Hahn et al. ( 6 ) documented that only one patient (out of three) had a 34% decrease from baseline, consistent with a partial response. Despite this, Chahoud et al. ( 7 ) reported that one of their patients had a complete response while the other had a partial response. They followed patients from 18 to 38 months, without having disease progression.

Despite all the described data, there was a multicenter phase II trial that started in 2016 and enrolled six patients. However, it was ended prematurely by poor accrual and no results were published.

Accordingly, the FDA approved Pembrolizumab to treat many tumor types that are MSI high, MMR deficient, or TMB high ( 4 ). In penile SCC, The National Comprehensive Cancer Network (NCCN) guidelines consider it as a salvage therapy option for those patients with TMB ≥10 ( 8 ) and MSI high tumors ( 5 ); despite this, it is still not clear when it is the best fit for these patients. Heterogeneity of tumor tissue and its dynamic nature over disease course, render another obstacle to getting uniform information ( 9 ). Higher TMB means that there is a higher frequency of gene mutations per coding area of a tumor’s genome ( 10 ). In the past years, there have been several efforts to assess biomarkers that predict response; however, results have not been consistent, and we could not fulfill the need for an ideal accurate biomarker.

Overall, 40-62% penile SCC express ≥1% PD-L1 on tumor or infiltrating immune cells ( 5 ); consequently, pembrolizumab might be a reasonable intervention. Still, with the currently available information, it is not possible to determine if this is completely accurate for penile SCC patients. Some authors have hypothesized that benefits occur irrespective of PD-L1 expression ( 11 ). Albeit statistically insufficient, this information supports previous evidence gathered from other urologic cancers. Other reports argue that high rates of MSI probably are related to DNA polymerase epsilon (POLE) and delta 1 (POLD) mutations rather than dMMR ( 12 ). To date, there is an unmet need for an ideal biomarker that predicts response to checkpoint inhibitors. We need to measure PD-L1 expression consistently and establish if TBM is a good surrogate marker for evaluating microsatellite instability. Furthermore, we could determine if POLE and POLD mutations are relevant.

In conclusion, we found a very scarce data, specifically only a few reports, but showing promising results for using pembrolizumab in advanced penile cancer patients. More trials need to be done to establish objective response and progression-free survival.
